# Exploring resting EEG correlates of age-related hearing difficulties

**DOI:** 10.3389/fnins.2026.1736209

**Published:** 2026-02-10

**Authors:** Cynthia R. Hunter

**Affiliations:** Speech Perception, Cognition, and Hearing Laboratory, Department of Speech-Language-Hearing: Sciences and Disorders, University of Kansas, Lawrence, KS, United States

**Keywords:** aging, alpha oscillations, EEG, hearing, hearing – disorders

## Abstract

**Background:**

Hearing loss is among the largest modifiable risk factors for dementia and has been associated with age-related decline in working memory capacity. How hearing loss might impact brain function is not yet well understood. Neural indices that are associated with age-related cognitive impairment and dementia can be derived from resting-state electroencephalography (rEEG). However, it is not known whether these are associated with hearing loss. The goal of the current study was to explore the relation of hearing ability to rEEG indicators of pathological brain aging.

**Design:**

Bivariate and partial correlations, controlling for age and education, of pure-tone-average (PTA) hearing loss, speech-in-noise ability, and self-reported hearing difficulty with oscillatory power in the alpha (8–13 Hz) and theta (4–7 Hz) bands as well as with individual alpha frequency (IAF) were explored in a sample of community-dwelling middle-aged and older adults (*N* = 62; 44 women; mean age = 67.18, range: 43–85). Correlations of the hearing factors with working memory capacity were also explored.

**Results:**

Correlations of rEEG measures with hearing ability measures were small-to-moderate (range: −0.05 to −0.25), with the only significant relation being that greater PTA hearing loss was associated with lower oscillatory power in the alpha band (bivariate rho(61) = −0.25, 95% CI: −0.47, −0.02; partial rho(59) = −0.23, 95% CI: −0.44, −0.01). Correlations of working memory with the hearing ability measures were also small-to-moderate in the bivariate analysis (range: −0.14 to −0.34) but weaker and not significant when age and education were controlled (range: −0.08 to −0.19).

**Conclusion:**

Poorer hearing ability in middle-aged and older adults may be associated with reduced resting alpha power. The small-to-moderate effect size of this relation was comparable in this sample to the known association of hearing loss with working memory capacity. Further work is warranted to investigate alpha power as a possible marker of changes to brain function associated with hearing loss.

## Introduction

Hearing loss is the third leading cause of disability in adults over 70 in the United States (Haile et al., 2024). It is also among the largest modifiable risk factors for dementia in high-income countries at the population level, where risk factor magnitude is scaled to reflect prevalence (Livingston et al., 2020, 2024). It is not yet known whether this link is causal. Hearing loss may be an early indicator of age-related neurological pathology, such that hearing loss and dementia share a common cause, or it may causally impact cognitive decline. Thus far, most evidence for links between neurological degeneration and hearing loss in humans has come from neuroimaging studies, which indicate that hearing loss in older adults is associated with structural changes such as reduced brain volume (An et al., 2023; Armstrong et al., 2019; Belkhiria et al., 2019; Eckert et al., 2019; Fitzhugh and Pa, 2022; Lin et al., 2014; Rigters et al., 2017; Wang et al., 2022), as well as altered functional connectively among brain regions (An et al., 2023; Fitzhugh et al., 2019; Xu et al., 2019; Zhu et al., 2020). Indicators of age-related cognitive decline may also be derived from resting EEG (rEEG), which is a non-invasive, relatively inexpensive, and quick-to-administer method of measuring brain activity that does not require people to perform potentially challenging tasks. As such, rEEG may offer useful indices of neural functioning for use in prospective studies aiming to elucidate links between hearing loss and age-related cognitive decline. Yet, it is unknown whether any rEEG indicators of age-related cognitive decline are associated with hearing loss. The goal of the current study was to explore correlations of hearing ability with known rEEG indicators of age-related cognitive decline in a cognitively healthy sample and to provide estimates of the magnitude of these relations in order to inform the design of future hypothesis-driven studies.

Particular neural oscillations, or rhythms, obtained through time-frequency analysis of EEG reflect activity in distinct neural networks that subserve key aspects of cognitive functioning. For example, alpha (8–12 Hz) oscillations are the most prominent rhythm of the brain especially at posterior sites, and appear to subserve attentional control throughout the cortex by tonically inhibiting processing that is not relevant to current goals (Jensen and Mazaheri, 2010; Klimesch, 2012). Theta (4–8 Hz) oscillations, particularly at fronto-medial sites, have been linked to working memory performance, memory consolidation, and cognitive control (Backus et al., 2016; Cavanagh and Frank, 2014; Eisma et al., 2021; Jensen and Tesche, 2002). There is also strong evidence that resting-state neural oscillations in the alpha and theta bands are altered in age-related cognitive decline and dementia. Lower alpha-band power and higher theta-band power have each been consistently associated with mild cognitive impairment (MCI) and neurodegenerative dementias including Alzheimer’s disease (Giustiniani et al., 2023; Jeong, 2004). Based on meta-analytic evidence that resting state alpha power decreases with increasing impairment from cognitive health to MCI and from MCI to dementia, resting alpha power has been suggested as a potential biomarker of early cognitive decline (Lejko et al., 2020). Among cognitively healthy adults, the literature on resting alpha and theta power and cognitive capacity is more mixed and sparse. Some studies with young adults have reported that lower resting alpha power is associated with poorer cognition (e.g., lower working memory capacity) (Mahjoory et al., 2019; Oswald et al., 2017). Older age has also been associated with lower resting alpha power (Babiloni et al., 2006; Park et al., 2024; Tröndle et al., 2023). However lower resting alpha power among cognitively healthy older adults has been associated with better working memory in at least one study (Borhani et al., 2021). Similarly, contrary to the pattern of increased theta power with pathological brain aging (e.g., Grunwald et al., 2007; Musaeus et al., 2018), greater resting theta power in healthy older adults has been associated with better cognition, suggesting different influences on theta power in healthy and pathological brain aging (Finnigan and Robertson, 2011; Vlahou et al., 2014). Another rEEG indicator of age-related cognitive health is the most prominent or peak frequency in the alpha band, which slows as adults age (Clark et al., 2004), is slower among young adults for those with poorer working memory (Clark et al., 2004; Klimesch, 1997), is slower among cognitively unimpaired older adults for those with poorer scores on various indicators of cognitive health including working memory (Chino et al., 2024; Clark et al., 2004), and also appears to slow with pathological brain aging (Garcés et al., 2013; López-Sanz et al., 2016; Puttaert et al., 2021; Stomrud et al., 2010).

In addition to being a risk factor for the development of cognitive impairment and dementia, hearing loss is also associated with poorer cognition among cognitively healthy older adults. Audiometric, pure-tone-average (PTA) hearing loss has been consistently associated with behavioral measures of cognitive functioning, including working memory (Lin et al., 2013; Loughrey et al., 2018). Among people with hearing loss, higher working memory capacity has also been consistently associated with better comprehension of speech in noisy backgrounds (for reviews see Akeroyd, 2008; Dryden et al., 2017). This may reflect an underlying association of working memory with higher-order auditory capabilities that are important for processing speech-in-noise (Humes, 2021). It may also reflect stronger ability among those with higher working memory capacity to effectively use effortful listening processes to understand speech-in-noise, such as holding chunks of uncomprehended speech in memory to await disambiguating information (Pichora-Fuller et al., 2016; Rönnberg et al., 2013). In turn, the added cognitive load of continual effortful listening in order to communicate may reduce neurocognitive capacity and increase the risk of developing symptoms of dementia (Wayne and Johnsrude, 2015).

Among cognitively healthy adults, various measures of neural oscillatory power have been shown to differ as a function of hearing loss when recorded during performance of cognitive tasks (Shende et al., 2024; Zhao et al., 2024) and also during speech-in-noise tasks (Alhanbali et al., 2022; Lai et al., 2023; Petersen et al., 2015; Price et al., 2019; Ryan et al., 2022), suggesting that hearing loss impacts neural functioning during task performance such as when listening to speech in noise. This is consistent with neuroimaging studies indicating compensatory processing in people with hearing loss during auditory tasks (Peelle and Wingfield, 2016; Slade et al., 2020). However, there appears to be no prior work examining whether rEEG indicators of age-related cognitive decline are associated with hearing loss. This would indicate whether neural functioning at rest is impacted by hearing loss. Such information would be useful for assessing associations of hearing loss with brain function that are not tied to performance of a task.

The current study explores the relation of hearing ability in a sample of cognitively healthy middle-aged and older adults to rEEG indicators of cognitive functioning, specifically alpha power, theta power, and alpha peak frequency. In parallel, the relation of hearing ability to working memory capacity was estimated in order to provide a point of reference with the association that many prior studies have used to quantify the link between hearing loss and age-related decline in cognitive functioning in cognitively unimpaired adults (Loughrey et al., 2018). Although prior studies on the relation of hearing loss to cognition have often relied on pure-tone average (PTA) hearing loss, in the current study the range of hearing ability metrics was broadened to include less widely available measures that may more closely track hearing-related functional difficulties. Reduced ability to understand speech in noisy environments and related difficulties with everyday functioning and participation in activities involving hearing are considered as central to the definition of hearing loss as PTA according to standards provided by the World Health Organization (2001), Humes (2019), and Stevens et al. (2013), and have long been considered central to understanding a person’s functional hearing abilities (Carhart and Tillman, 1970; Crandell, 1991; National Academies of Sciences, Engineering, and Medicine, 2025; Wilson and McArdle, 2005). The current study includes PTA hearing loss as well as an objective measure of speech-in-noise perception and surveys of self-reported hearing difficulties as measures of hearing ability. Neither the direction nor magnitude of correlations among the hearing ability and rEEG factors was known, and as such it was not deemed reasonable to do power analysis or make directional predictions. This lack of knowledge motivated the current study, in order to provide such estimates for future, hypothesis-driven studies.

## Materials and methods

### Participants

Data was analyzed from 68 middle-aged and older adults (mean age = 67.18, range: 43–85; 44 women, 18 men) who participated in a larger study. All participants were native English speakers who had a normal tympanogram in their better-hearing ear and passed a screener for cognitive impairment (MoCA ≥21; cutoff chosen for specificity based on normative date, i.e., to support inclusion of cognitively unimpaired adults 60–90 years of age) (Rossetti et al., 2011; Sachs et al., 2022). Participants all gave written informed consent and were paid $15 for each hour of participation, in accordance with procedures approved by the appropriate Institutional Review Board.

### Measures

The experiment took place over two sessions. Hearing assessment took place at the beginning of the first session, and rEEG data was collected at the beginning of the second session. Mean duration between sessions was 15 days. The relevant tasks from each session are summarized below.

#### Session 1

##### Hearing difficulty questionnaires

Participants completed the following self-report questionnaires about hearing difficulties: Speech and Spatial Qualities of Hearing Scale (SSQ) (Gatehouse and Noble, 2004), Revised Hearing Handicap Inventory (RHHI) (Cassarly et al., 2020), Listening Effort Assessment Scale (EAS) (Alhanbali et al., 2017), and Vanderbilt Fatigue Scale for Adults (VFS-A-40) (Hornsby et al., 2021). Responses from each survey were scored such that larger numbers indicate greater hearing difficulty.

##### Hearing assessment

A standard Hughson-Westlake procedure using a Maico MA-42 audiometer with insert earphones was used to determine pure-tone thresholds for frequencies ranging from 0.25 to 8 kHz. The four-frequency (0.5, 1, 2, 4 kHz) pure tone average (4fPTA) air-conduction threshold in the better-hearing ear was used to quantify degree of hearing loss. A Maico TouchTymp MI-24 tympanometer was used to obtain tympanograms.

##### Speech detection threshold

This task was used to set individualized overall presentation levels for the Words in Noise (WIN) Test (see below), such that speech-in-noise testing was done with audibility roughly equated across participants. The 50 percent threshold for detecting a 2-s segment of the background babble used in the listening task in Session 2 was determined using a 1-up, 1-down adaptive psychophysical staircase procedure with a two-interval forced choice task.

##### Words in noise test

A computerized version of the WIN Test was implemented using pre-recorded spoken sentence and noise samples (Disc 4.0 of Speech Recognition and Identification Materials, issued by the Department of Veterans Affairs). In this test, five sentences are presented at each of seven signal-to-noise ratios (SNR) from 24 to 0 dB in 4-dB decrements. Further details of the WIN Test may be found in Wilson and McArdle (2007). Background noise was presented at the lower of 75 dB SPL or 40 dB SL based on the SDT threshold (see above). The stimuli were presented over loudspeakers positioned at ear height 60 degrees to participants frontal left and right. In order to approximate typical listening conditions for participants who used and did not use hearing aids, participants were asked to wear their own hearing aids for all sessions of the experiment (excluding audiometry and tympanometry) if they wore hearing aids 50 percent or more of the time in their daily life. Five participants (4 women) wore a hearing aid (4 bilateral). The estimated signal-to-noise ratio at which each participant scored 50 percent correct (SNR50) on the WIN Test was used to quantify speech-in-noise performance.

##### Working memory

A computerized reading span task with established reliability and convergent validity was used to measure working memory capacity (Lewandowsky et al., 2010). The task includes a processing and a memory component. The processing task was to judge whether sentences presented on a computer screen (e.g., “An eagle has a fin.”) were true or false by pressing a button on the keyboard. Following each true or false response, a consonant was presented on the computer screen. After a variable number of sentence/consonant pairs ranging from four to seven, participants were prompted to recall the consonants in order. Working memory capacity was quantified as the proportion of items for which all the elements were recalled correctly, as follows. Trials on which all consonants were recalled correctly in order were scored as “1” and other trials as “0.” The mean score across trials was taken as the measure of working memory capacity [“all-or-nothing unit scoring”—see Lewandowsky et al. (2010) for discussion].

#### Session 2

##### Resting EEG

Prior to beginning a longer listening task, automated spoken instructions guided participants through two minutes of eyes-closed and two minutes of eyes-open EEG. Two minutes of resting EEG appears to be a sufficient duration for measuring oscillatory power in the alpha band with good-to-excellent reliability and in the theta band with fair reliability in older adults (Popov et al., 2023). Order of eyes-closed and eyes-open presentation was randomized.

### EEG recording and preprocessing

Electroencephalogram (EEG) was recorded with a 64-channel ActiCAP snap cap (Easycap GmbH) using an actiCHAMP amplifier (Brain Products, Inc.) and Brain Vision Recorder software. Data was recorded with Fz as the reference electrode at a sampling rate of 1,000 Hz and a low-pass filter of 280 Hz. Electrode impedances were set to 25 kOhm for the ground electrode and below 60 kOhm for other electrodes, as per the manufacturer’s instructions.

Post-acquisition, resting EEG data were analyzed using the EEGLAB toolbox (Delorme and Makeig, 2004), an analysis toolbox for Matlab, including in-house routines written to run in EEGLAB. The data was digitally high-pass filtered 0.1 Hz and low-pass filtered at 100 Hz. An automated function was used to remove line noise (CleanLine plugin for EEGLAB). The continuous data was segmented into 1-s epochs for preprocessing. Epochs were visually inspected to identify bad channels, which were removed – the most channels removed for any participant was 18 and the median removed was three. Epochs with gross electro-ocular and/or electromyographic artifacts were removed using visual inspection (a mean of 8.16 percent of epochs were removed (*SD* = 7.77)). Data from six participants (5 female) with greater than 20 percent of epochs rejected were excluded from further analysis. After artifact rejection, any missing channels were spherically interpolated and data were re-referenced to an average reference.

Spectral power was estimated using the EEGLAB function “std_precomp” to compute the power spectrum with Hamming-windowed data segments (Delorme and Makeig, 2004). The default parameters of “fft” for “specmode” and “off” for “logtrials” were used, such that the power spectrum was computed as the square of the amplitude of the Fast Fourier Transform (FFT). For statistical analysis, mean spectral power was extracted in the alpha (8–13 Hz) frequency range at posterior sites (CPz, CP1, CP2, CP3, CP4, Pz, P1, P2, P3, P4, P5, P6, POz, PO3, PO4, Oz, O1, O2) and in the theta (4–7 Hz) frequency range at frontomedial sites (Fz, F1, F2, FCz, FC1, FC2). These values were then averaged across electrode to yield a single value for each participant and condition (eyes closed, eyes open).

**Figure 1:**
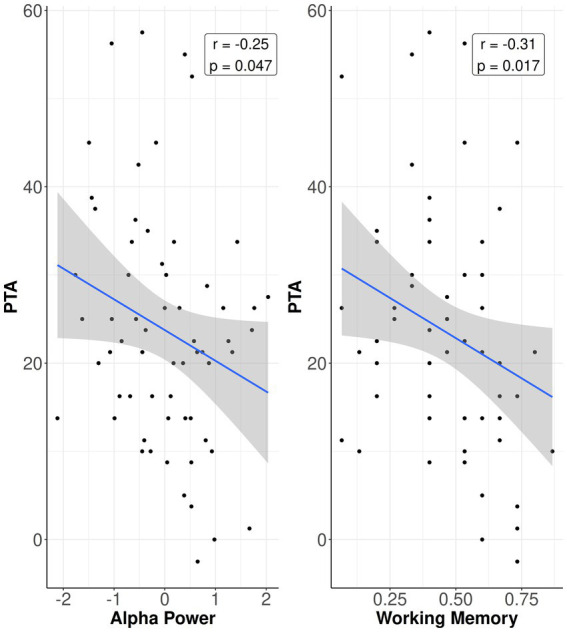
Correlations. Relation between PTA hearing loss and alpha power (left) and working memory capacity (right). Data points represent individual observations. Blue line shows linear regression line between factors; shaded area shows 95 percent confidence interval.

Individual alpha frequency (IAF) was estimated using the “restingIAF” Matlab function provided by Corcoran et al. (2018), which provides two indices of individual alpha frequency: peak alpha frequency and center of gravity. The peak alpha frequency estimates the location of a singular, prominent spectral peak. The center of gravity estimates the mean frequency from a weighted average of alpha-band power, and may thus be more robust in the absence of a single spectral peak in the alpha band. Both estimates are derived from power spectral density smoothed with a Savitsky-Golay filter. Following procedures described by Corcoran and colleagues, data was down-sampled to 250 Hz prior to analysis and IAF was estimated for the eyes-closed condition only (which is expected to elicit more pronounced alpha spectral peaks than eyes-open conditions) and from a subset of posterior channels where alpha power was most pronounced (Pz, P1, P2, P3, P4, P5, P6, POz, PO3, PO4, Oz, O1, O2) (see Figure 1). Optional parameters followed Cocoran et al., with the a minimum of 3 channel estimates to be resolved in order to calculate the IAF, a frame width of 11, and a polynomial order of 5. Two optional parameters were altered: (1) the minimum height difference distinguishing a primary peak from competing peaks was lowered from 20 percent to 15 percent, and (2) the lower bound of the alpha peak search window was lowered from 7 to 6 Hz (for a search window of 6–13 Hz) in order to accommodate age-related slowing of alpha power in older adults (Klimesch, 1999). Estimates were obtained for 60 out of 62 participants.

### Statistical analyses

Both bivariate and partial correlations, controlling for age and education, were used to provide estimates of relations among PTA hearing loss, speech-in-noise ability, self-reported hearing difficulty, alpha power, theta power, IAF, and working memory capacity. Given that a number of variables were not normally distributed (e.g., hearing variables were positively skewed) and/or contained outliers, that the hearing difficulty surveys originated from ordinal rather than interval data, and that there was no *a priori* reason to assume a specifically linear rather than simply monotonic relation between variables, Spearman’s correlation was used for all tests in order to maintain a consistent approach for all point estimates and confidence intervals. If a participant was missing data in one or both variables, the participant was dropped from that correlation. Given the exploratory nature of the study, no correction was made for multiple comparisons.

All analyses were performed using R Statistical Software (v4.5.2; R Core Team, 2025) using the RStudio environment (Posit team, 2025). For pairwise correlations, estimates and significance levels were computed using the “cor.test” function in base R, and confidence intervals were obtained using the “spearman.ci” function from the RVAideMemoire package (Herve, 2025). For partial correlations controlling for age and education, the “partial_Spearman” function from the PResiduals package (Liu et al., 2020) was used to obtain estimates, significance levels, and confidence intervals.

The education variable was reported by participants categorically (e.g., “some college,” “master’s degree”; see Table 1) and was converted to corresponding years of education for analysis. Inter-correlations among the hearing difficulty surveys were all large in magnitude (all Spearman rho > 0.50). Therefore, the surveys were combined into a single “self-reported hearing difficulty” factor by averaging the z-scores of the individual surveys. Similarly, spectral power in eyes-closed and eyes-open conditions was highly correlated for both alpha and theta bands (both Spearman rho >0.65). Therefore, power in each frequency band was combined into a single alpha power factor and a single theta power factor by averaging the respective z-scores. The two estimates of IAF, peak alpha frequency and center of gravity, were also highly correlated (Spearman rho >0.85) and were averaged to create a single IAF factor.

**Table 1:** Participant demographics.

Measure	All (*n* = 62)	Women (*n* = 44)	Men (*n* = 18)
Age, years, mean (SD)	67.18 (9.04)	66.86 (9.16)	67.94 (8.95)
Education, *n* (%)			
Some college	6 (10)	4 (9)	2 (11)
Vocational or technical degree	2 (3)	1 (2)	1 (6)
College degree	18 (29)	14 (32)	4 (22)
Some graduate school	4 (7)	3 (7)	1 (6)
Master’s degree	25 (40)	18 (41)	7 (39)
Doctorate/Medical degree	7 (11)	4 (9)	3 (17)
Race, *n* (%)			
White	60 (97)	43 (98)	17 (94)
Prefer not to say	2 (3)	1 (2)	1 (6)
Ethnicity, *n* (%)			
Hispanic/Latino	4 (7)	3 (7)	1 (6)
Not Hispanic Latino	56 (90)	40 (91)	16 (89)
Prefer not to say	2 (3)	1 (2)	1 (6)
MoCA, *n* (%)			
21–22	3 (5)	1 (2)	2 (11)
23–25	8 (13)	6 (14)	2 (11)
26–30	51 (82)	37 (84)	14 (78)
PTA, dB HL, mean (SD)	23.75 (13.52)	22.70 (12.22)	26.32 (16.39)
SNR50, dB, mean (SD)	7.79 (3.30)	7.16 (2.44)	9.34 (4.55)
Hearing survey, z-score, mean (SD)	−0.04 (0.86)	−0.13 (0.87)	0.17 (0.83)
Working memory, percent, mean (SD)	45.89 (20.13)	45.89 (19.31)	45.88 (22.72)

To support consideration of the influence of biological sex as recommended by the National Institutes of Health (2015), all correlations disaggregated by sex are provided as Supplementary materials.

## Results

### Audiometry

**Figure 2:**
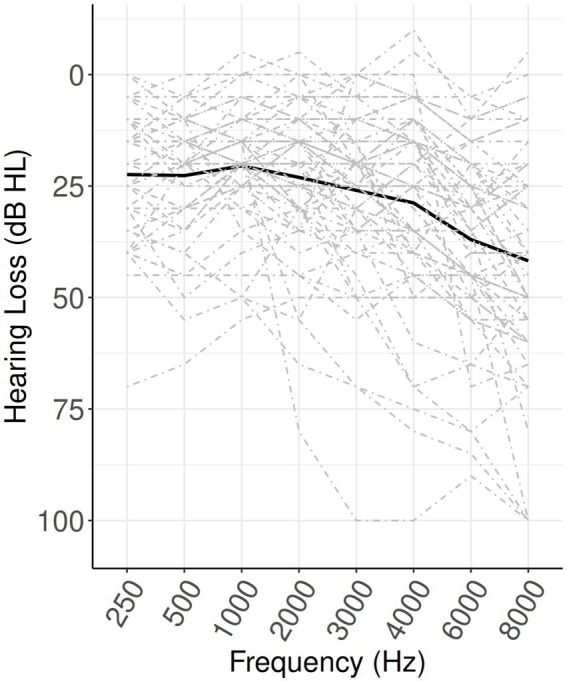
Audiograms. Bold line shows mean pure-tone hearing thresholds. Dashed lines show individual participants.

Better-ear audiograms are shown in Figure 2. Participants had a range of hearing abilities and, on average, hearing loss that was greater at higher frequencies. According to World Health Organization proposed criteria based on the better-ear 4fPTA (Stevens et al., 2013; Olusanya et al., 2014), hearing loss levels in the sample were as follows: 34 percent no hearing loss, 48 percent mild, 11 percent moderate, and 7 percent moderately severe.

### Bivariate correlations

Bivariate correlations and corresponding confidence intervals are shown in Table 2 [disaggregated by sex in Supplementary Table 1 (women) and Supplementary Table 2 (men)]. From Table 2 it can be seen that correlations of rEEG measures with the hearing ability measures were small-to-moderate (range: −0.10 to −0.25), with the only significant relation being that greater PTA hearing loss was associated with lower alpha power (rho(60) = −0.253, *p* = 0.047). The correlations of working memory to hearing ability were comparable in magnitude (range: −0.14 to −0.34), with significant associations of lower working memory capacity with greater PTA hearing loss (rho(58) = 0.307, *p* = 0.017) and higher (worse) speech-in-noise ability (rho(57) = 0.342, *p* = 0.008). Figure 1 plots the relation of PTA to alpha power and to working memory capacity.

**Table 2:** Pair-wise correlations.

Measure	1	2	3	4	5	6	7
1. Age	–						
2. PTA	0.401** [0.139, 0.607]	–					
3. Speech-in-Noise	0.318* [0.040, 0.543]	0.649*** [0.481, 0.781]	–				
4. Self-report	0.077 [−0.186, 0.326]	0.500*** [0.274, 0.677]	0.482** [0.275, 0.653]	–			
5. Alpha	−0.073 [−0.332, 0.177]	−0.253* [−0.473, −0.021]	−0.169 [−0.436, 0.074]	−0.210 [−0.444, 0.055]	–		
6. Theta	−0.126 [−0.391, 0.148]	−0.218 [−0.451, 0.029]	−0.188 [−0.437, 0.095]	−0.250 [−0.487, 0.002]	0.518*** [0.282, 0.694]	–	
7. IAF	−0.113 [−0.356, 0.144]	−0.102 [−0.353, 0.141]	−0.156 [−0.375, 0.100]	−0.162 [−0.403, 0.092]	−0.234 [−464, 0.018]	−0.094 [−0.347, 0.164]	–
8. Working Memory	−0.266* [−0.516, −0.008]	−0.307* [−0.528, −0.041]	−0.342** [−0.547, −0.104]	−0.139 [−0.368, 0.095]	0.055 [−0.188, 0.277]	0.034 [−0.237, 0.290]	0.288* [0.044, 0.510]

*p < 0.05; **p < 0.01; ***p < 0.001.

Working memory was also related to IAF, such that higher working memory capacity was associated with faster IAF (rho (56) = 0.288, *p* = 0.029). It can also be seen from Table 1 that the hearing ability measures were intercorrelated (range: 0.48 to 0.65), and that alpha and theta power were positively associated (rho (60) = 0.518, *p* < 0.001). Finally, older chronological age was associated with greater PTA hearing loss (rho (60) = 0.401, *p* = 0.001), poorer speech-in-noise ability (rho (57) = 0.318, *p* = 0.014), and smaller working memory capacity (rho (58) = −0.266, *p* = 0.040), but was not significantly related to any of the rEEG measures (rho range: −0.07 – −0.13).

### Partial correlations

Partial correlations controlling for age and education are shown in Table 3 [disaggregated by sex in Supplementary Table 3 (women) and Supplementary Table 4 (men)]. From Table 3 it can be seen that correlations of rEEG and hearing ability measures remained small-to-moderate (range: −0.05 to −0.233), with the only significant relation again being that greater PTA hearing loss was associated with lower alpha power (rho(58) = −0.233, *p* = 0.045). Correlations of working memory and hearing ability were reduced compared to the bivariate correlations (range: −0.08 to −0.19) and none were significant.

**Table 3:** Partial correlations, controlling for age and education.

Measure	1	2	3	4	5	6
1. PTA	–					
2. Speech-in-Noise	0.560*** [0.348, 0.718]	–				
3. Self-report	0.497*** [0.274, 0.669]	0.482*** [0.296, 0.634]	–			
4. Alpha	−0.233* [−0.438, −0.006]	−0.080 [−0.334, 0.182]	−0.195 [−0.431, 0.065]	–		
5. Theta	−0.260 [−0.493, 0.007]	−0.148 [−0.395, 0.117]	−0.234 [−0.468, 0.032]	0.497*** [0.256, 0.680]	–	
6. IAF	−0.053 [−0.303, 0.205]	−0.119 [−0.374, 0.153]	−0.157 [−0.399, 0.106]	−0.262* [−0.490, −0.001]	−0.115 [−0.372, 0.157]	–
7. Working Memory	−0.180 [−0.410, 0.071]	−0.191 [−0.422, 0.064]	−0.084 [−0.328 0.170]	−0.045 [−0.289, 0.204]	−0.086 [−0.345, 0.186]	0.291* [0.039, 0.508]

*p < 0.05; **p < 0.01; ***p < 0.001.

With age and education controlled, higher working memory capacity remained related to faster IAF (rho (54) = 0.291, *p* = 0.024), hearing ability measures remained intercorrelated (range: 0.48 to 0.56), and alpha and theta power remained positively associated (rho (58) = 0.497, *p* < 0.001). In addition, the relation of alpha power and IAF emerged as significant (rho (58) = −0.262, *p* = 0.049), such that higher alpha power was associated with lower IAF.

## Discussion

This exploratory study provides novel information about the relation of hearing difficulties in middle and older age to rEEG indicators of age-related cognitive decline. Small-to-moderate, significant associations were observed between hearing abilities and alpha power in this sample, such that poorer hearing ability was associated with reduced resting alpha power. These associations were comparable in magnitude to the well-known association of hearing loss with working memory capacity. Although the associations of hearing abilities with working memory did not remain significant with age and education controlled, poorer hearing loss remained associated with reduced alpha power.

The relation of hearing loss to resting alpha power observed here is similar in magnitude to a recent report of a correlation of PTA hearing loss to alpha power in an active design in which alpha power was measured before each trial in a speech-in-noise task in older adults (Alhanbali et al., 2022). The current finding also bears some similarity to a report of lower alpha power in older adults with than without hearing loss during a speech-in-noise task (Lai et al., 2023). Lai and colleagues also found that older adults with hearing loss exhibited altered modulation of brainstem speech processing as a function of cortical alpha state. An influence of hearing loss on alpha power in a speech-in-noise task was also observed by Petersen and colleagues (2015), who reported a “breakdown” in alpha power modulation by signal-to-noise ratio in those older listeners with the most pronounced hearing loss during an auditory memory task. The current findings add to this literature by suggesting that even during a period of rest before any active task is performed, alpha power is reduced among aging adults with hearing loss. These results may also be taken as a cautionary note for interpretation of effects of hearing loss on alpha power in active tasks, when a task-free rest period is used as a baseline. If resting alpha power varies as a function of hearing loss, subtracting resting power from power during a task could reflect or mirror differences into the active-recording interval that originate in the baseline period. Finally, the current findings provide a basis for future work to investigate the hypothesis that there is a small-to-moderate association between lower resting alpha power and greater degree of hearing loss in cognitively healthy middle-aged to older adults.

The relation of hearing loss to working memory in cognitively unimpaired older adults is a well-documented link that has been repeatedly observed in prior studies, and that is presumed to have similar underlying cause(s) as the link between hearing loss and pathological brain aging (Loughrey et al., 2018). The similar magnitude of the relations of hearing loss with working memory and alpha power in the current sample suggests that resting alpha power may also index a key aspect of brain function that is affected by hearing loss. Consistent with prior research, working memory was related to IAF (Chino et al., 2024; Clark et al., 2004); however, working memory did not appear related to resting alpha power (see Tables 1, 2). As such, the relation of hearing loss with alpha power in the current sample may indicate a separate pathway by which hearing loss and brain function are related in middle-aged to older adults.

If confirmed by hypothesis-driven research, the link between hearing loss and resting alpha power could potentially be leveraged in future research to better characterize the association of hearing loss and accelerated age-related decline in neural functioning. For example, given that higher resting alpha power has been taken as an index of better ability of the brain to maintain a strong attentional filter (Klimesch, 2012), reduced alpha power in people with hearing loss might signal reduced capacity to inhibit attention to distracting stimuli and/or reduced attentional readiness. Resting alpha power is also an indicator of cortical arousal, with lower power corresponding to higher cortical excitability (Barry et al., 2011; Barry and De Blasio, 2017; Klimesch, 1999). Elevated cortical excitability may mediate some age-related deficits in cognitive performance (Cespón et al., 2022; Cardone et al., 2021). In line with this, decreases in resting alpha power have been associated with changes in cholinergic basal forebrain system function (Holschneider et al., 1998; Yang et al., 2022), which in turn have been associated with pathological brain aging (Auld et al., 2002; Lauterborn et al., 2021), Further, among older adults with MCI and Alzheimer’s dementia, lower resting alpha power has been associated with reduced gray matter volume and poorer cognition, suggesting that reduced posterior alpha power can be a biomarker of pathological brain aging (Babiloni et al., 2013). Possibly, reduced alpha power in aging adults with hearing loss could be an indicator of changes to brain function that underlie early subclinical stages of a type of pathological brain aging that is linked with hearing loss. Alternatively, such an association could reflect compensatory or adaptive rather than pathological changes to brain function associated with hearing loss. This would be consistent with a prior study in which lower resting alpha power among cognitively healthy older adults was associated with better cognitive performance (Borhani et al., 2021).

A limitation of the current study is that given the wide age range of participants, there may have been differences associated with age that were not directly statistically controlled, such as physical and psychological health, level of social engagement, and lifestyle differences (e.g., employed versus retired). Any such differences could have obscured bivariate correlations, and could possibly have been unintentionally factored out of the partial correlations that statistically controlled for age. Another limitation relates to the range of hearing abilities in the sample. Most participants with hearing loss had a mild degree of hearing loss, and results could have differed if a greater portion of the sample had a greater extent of hearing loss.

The current results provide initial estimates of the relation of hearing difficulties to rEEG indicators of cognitive function. Even small correlations are “important” in this context – from the high prevalence of hearing loss in older adults, it follows that even small associations with hearing loss in this population will be present in a substantial number of individuals. In the current study, small-to-moderate associations of hearing difficulties with alpha power were observed, and were comparable in magnitude to the known association of hearing loss with working memory capacity. Future, hypothesis-driven studies are warranted to provide more precise estimates of the relation of hearing difficulties to resting alpha power.

## Data Availability

The datasets presented in this study can be found in online repositories. The names of the repository/repositories and accession number(s) can be found at: Open Science Framework, https://doi.org/10.17605/OSF.IO/Z8ADG.
