# Supplementary material for: Exploring resting EEG correlates of age-related hearing difficulties
Source: Front Neurosci. 2026 Feb 10;20:1736209. doi: 10.3389/fnins.2026.1736209 (PMC12947132; doi:10.3389/fnins.2026.1736209)
Supplement: Supplementary file 1 [file Data_Sheet_1.PDF]

Supplementary Table 1.

Pair-wise correlations for women.

	1	2	3	4	5	6	7
**1. Age**	__						
**2. PTA**	.300	__					
	[-.030, .581]						
**3. Speech-in-**	.150	.618***	__				
**Noise**	[-.164, .450]	[.352, .805]					
**4. Self-report**	-.017	.500***	.394*	__			
	[-.319, .270]	[.228, .707]	[.085, .634]				
**5. Alpha**	-.155	-.334*	-.322*	-.263	__		
	[-.439, .144]	[-.575, -.071]	[-.587, -.025]	[-.550, .052]			
**6. Theta**	-.104	-.151	-.133	-.243	.460**	__	
	[-.384, .197]	[-.434, .170]	[-.407, .202]	[-.524, .071]	[.172, .672]		
**7. IAF**	-.036	.032	-.138	-.082	-.281	-.186	__
	[-.338, .282]	[-.259, .339]	[-.459, .194]	[-.342, .220]	[-.541, .029]	[-.506, .136]	
**8. Working**	-.370*	-.309*	-.301	-.063	.139	-.022	.366*
**Memory**	[-.624, -.038]	[-.590, -.010]	[-.532, .002]	[-.336, .245]	[-.155, .416]	[-.366, .301]	[.074, .591]

* p < .05; ** p < .01; *** p < .001

Supplementary Table 2.

Pair-wise correlations for men.

	1	2	3	4	5	6	7
**1. Age**	__						
**2. PTA**	.522*	__					
	[-.038, .846]						
**3. Speech-in-**	.665**	.663**	__				
**Noise**	[.237, .923]	[.244, .873]					
**4. Self-report**	.238	.539*	.513*	__			
	[-.273, .653]	[.066, .828]	[.100, .776]				
**5. Alpha**	.172	-.081	.274	-.022	__		
	[-.383, .623]	[-.520, .371]	[-.254, .698]	[-.517, .482]			
**6. Theta**	-.235	-.277	-.207	-.282	.414	__	
	[-.686, .358]	[-.694, .281]	[-.727, .368]	[-.659, .202]	[-.116, .794]		
**7. IAF**	-.251	-.321	-.068	-.251	-.143	.148	__
	[-.752, .258]	[-.659, .110]	[-.494, .394]	[-.701, .310]	[-.605, .361]	[-.361, .569]	
**8. Working**	.035	-.335	-.505*	-.404	-.234	.107	.154
**Memory**	[-.497, .537]	[-.697, .172]	[-.852, -.033]	[-.786, .104]	[-.608, .284]	[-.422, .594]	[-.346, .652]

$* p < .05$; $** p < .01$; $*** p < .001$

Supplementary Table 3

Partial correlations for women, controlling for age and education.

	1	2	3	4	5	6
**1. PTA**	__					
**2. Speech-in-Noise**	.632*** [.367, .802]	__				
**3. Self-report**	.537*** [.283, .720]	.414** [.145, .626]	__			
**4. Alpha**	-.273 [-.518, .013]	-.248 [-.521, .071]	-.251 [-.528, .074]	__		
**5. Theta**	-.184 [-.460, .124]	-.134 [-.419, .175]	-.239 [-.516, .083]	.430** [.137, .653]	__	
**6. IAF**	.055 [-.260, .360]	-.143 [-.458, .204]	-.089 [-.367, .204]	-.331 [-.601, .007]	-.213 [-.511, .131]	__
**7. Working Memory**	-.161 [-.425, .129]	-.157 [-.433, .147]	-.044 [-.333, .253]	.001 [-.312, .315]	-.056 [-.382, .283]	.384* [.064, .632]

* p < .05; ** p < .01; *** p < .001

Supplementary Table 4

Partial correlations for men, controlling for age.

	1	2	3	4	5	6
**1. PTA**	__					
**2. Speech-in-Noise**	.190<br>[-.332, .624]	__				
**3. Self-report**	.419<br>[-.123, .768]	.480*<br>[.037, .765]	__			
**4. Alpha**	-.332<br>[-.694, .164]	.364<br>[-.099, .698]	-.072<br>[-.535, .423]	__		
**5. Theta**	-.326<br>[-.688, .167]	-.060<br>[-.523, .431]	-.264<br>[-.597, .147]	.419<br>[-.158, .782]	__	
**6. IAF**	-.208<br>[-.614, .285]	.029<br>[-.406, .453]	-.240<br>[-.666, .303]	-.121<br>[-.561, .372]	.101<br>[-.344, .509]	__
**7. Working Memory**	-.463<br>[-.794, .081]	-.656**<br>[-.864, -.258]	-.381<br>[-.725, .114]	-.230<br>[-.608, .234]	.142<br>[-.326, .553]	.138<br>[-.332, .554]

* p < .05; ** p < .01; *** p < .001
